# Duration of fever and serious bacterial infections in children: a systematic review

**DOI:** 10.1186/1471-2296-12-33

**Published:** 2011-05-16

**Authors:** Gijs Elshout, Miriam Monteny, Johannes C van der Wouden, Bart W Koes, Marjolein Y Berger

**Affiliations:** 1 Erasmus MC, Department of General Practice, PO Box 2040, 3000 CA Rotterdam, The Netherlands; 2 University Medical Center Groningen, University of Groningen, Department of General Practice, PO Box 196, 9700 AD Groningen, The Netherlands

## Abstract

**Background:**

Parents of febrile children frequently contact primary care. Longer duration of fever has been related to increased risk for serious bacterial infections (SBI). However, the evidence for this association remains controversial. We assessed the predictive value of duration of fever for SBI.

**Methods:**

Studies from MEDLINE, Embase and Cochrane databases (from January 1991 to December 2009) were retrieved. We included studies describing children aged 2 months to 6 years in countries with high Haemophilus influenzae type b vaccination coverage. Duration of fever had to be studied as a predictor for serious bacterial infections.

**Results:**

Seven studies assessed the association between duration of fever and serious bacterial infections; three of these found a relationship.

**Conclusion:**

The predictive value of duration of fever for identifying serious bacterial infections in children remains inconclusive. None of these seven studies was performed in primary care. Studies evaluating the duration of fever and its predictive value in children in primary care are required.

## Background

Fever is very common among young children and a frequent reason for parents to contact primary care [1]. Febrile children usually have self-limiting viral infections, and serious infections in need of medical intervention are rare. In primary care, clinical markers are the most appropriate evaluation tools in febrile children. In some studies, the duration of fever prior to presentation has been shown to be a predictor of serious bacterial infection (SBI) [2-6]. However, the evidence for this association remains a subject of discussion [7]. For example, in the practice guideline for the management of febrile children in primary care, the Dutch College of General Practitioners (NHG) recommends that children with more than three days of fever at presentation should be seen by a general practitioner (GP) [7]. In contrast, the NICE guideline for feverish illness in children in the UK states that duration of fever should not be used to predict the likelihood of serious illness, other than Kawasaki disease [8]. Both guidelines base these recommendations on studies performed in secondary and tertiary care, which may not be applicable for primary care settings.

Since the introduction of the *Haemophilus influenzae *type b (Hib) conjugate vaccine during the last two decades, the prevalence of Hib-induced infections has decreased [9]. This might have consequences for the association between duration of fever and SBI. Gaining more insight into the course of fever in the post-Hib era is essential for the evaluation and management of febrile children in primary and secondary care.

Therefore, we conducted a systematic review of studies on duration of fever in children aged two months to six years, in the post-Hib era. We aimed at answering the question: what is the association between duration of fever and an SBI in febrile children?

## Methods

### Identification and selection of the literature

A systematic search of the literature was made from January 1991 to December 2009 in the MEDLINE, Embase and Cochrane databases. Since Hib vaccination was not widely distributed before 1991 [10], the search was restricted to the years after 1990. Sensitive search strategies ('clinical queries') were used for prognostic studies [11], diagnostic studies [12] and randomized controlled trials (RCTs) [13]. The following keywords and MeSH-headings were used: 'fever', 'preschool child', 'infant', 'childhood', 'course*', 'duration', 'disease', 'infection', 'bacterial infection', 'bacterial infections', 'serious bacterial infection*', 'mortality', 'child hospitalization', and 'hospitalization' (see additional file 1, Table S1). Reference lists of selected publications were checked to identify additional relevant publications.

To identify eligible studies, titles and abstracts resulting from the search strategy were screened independently by two teams of reviewers (MM/GE and MYB/JCvdW). Studies had to meet the following criteria:

1) The design of the study was a prospective cohort study, cross-sectional study or RCT.

2) The majority of participants were children aged two months to six years (or an identifiable and separately analyzed subgroup of at least ten children aged two months to six years).

3) Enrolment occurred in a country with adequate Hib vaccination coverage, i.e. ≥ 80% according to WHO/UNICEF estimates [10], during at least 50% of the enrolment period.

4) The outcome measure was duration of fever (prior to enrolment) as prognostic factor for SBI.

5) In case of SBI, eligible diagnoses included bacteremia, sepsis, bacterial meningitis, bacterial pneumonia, infectious arthritis, osteomyelitis, cellulitis, soft tissue infection, pyelonephritis, urinary tract infection, bacterial gastroenteritis, tonsillitis, or otitis media.

Studies focusing on immunocompromized children or fever syndromes were excluded. Studies in countries outside Europe, North America, Australia or New Zealand were excluded, because the etiology, prevalence and presentation of febrile illnesses differ significantly in these countries.

### Data extraction

Two teams of reviewers (MM/GE and MYB/JCvdW) independently extracted data from the selected studies using standardized forms. The extracted data concerned design, setting, study population, outcome measures and prognostic factors.

### Quality assessment

Two teams of reviewers (MM/GE and MYB/JCvdW) assessed the methodological quality of the studies independently, by means of a modified version of the criteria list for prognostic studies as developed by Hayden et al. [14] Since cross-sectional studies were also included, we added an item concerning the independent assessment of duration of fever and SBI diagnosis. The list consisted of 22 items (Table 1) that were scored positive (+), negative (-), unclear (?) or not applicable (NA). Disagreement between the reviewers was discussed in a consensus meeting.

**Table 1:** Items included in the methodological quality assessment.

*Study participation*	
1	Setting of recruitment is described
2	Moment of identification is described and equal for all included children (inception cohort)
3	Percentage participation of eligible children is described
4	Inclusion and exclusion criteria are described and age, fever and relevant co-morbidity are reported
5	Baseline study sample is described for key characteristics age and sex
*Study attrition*	
6	Number of loss to follow-up in cohort study/RCT is <20%, or the number missing for analysis (the difference between number included and number analyzed) in cross-sectional studies is <20%
7	Reasons for loss to follow-up/missing for analysis are provided
8	Key characteristics (at least age and sex) of participants lost to follow-up/missing for analysis do not differ significantly from the study sample
*Prognostic factor measurement*	
9	Prognostic factor duration of fever: method of measurement is described and valid (thermometer)
10	Prognostic factor duration of fever: duration prior to presentation is described
11	Prognostic factor SBI: definition of diagnosis is described and valid
12	If continuous variables are used, they are reported as continuous variables or appropriate cut-off points (not data dependent) are used
*Outcome measurement*	
13	A clear definition of the outcome (duration of fever, SBI or hospitalization) is provided
14	Method and setting of outcome measurement are the same for all study participants
15	SBI was assessed independently from the assessment of fever
*Confounding measurement and account*	
16	Antipyretics use before and/or during the study is assessed and reported
17	Antibiotics use before and/or during the study is assessed and reported
18	Level of illness is measured and measurement method is appropriate (e.g. Yale score) and the same for all children
19	The potential confounders antipyretics use, antibiotics use and illness level are accounted for in the study design or analysis
*Analysis*	
20	There is data presentation of the prognostic factors duration of fever and/or SBI
21	The association of prognostic factor and outcome is given in percentages or means/medians, or in OR/RRs with confidence interval/SD, or calculation of these measures is possible
22	A multivariate model is used in the analysis

Inter-assessor agreement of the methodological quality assessment was calculated using kappa scores [15]. The total quality score for each study was calculated by counting all positively scored criteria (maximum 22) and dividing this number by the number of applicable items. High quality was defined as a score of 50% or higher.

### Analysis

The studies included in this review were considered too heterogeneous (regarding setting, definition of fever and of SBI, and presentation of the results) to pool the data. Therefore, a best-evidence synthesis was used to summarize the value of prognostic factors. Four levels of evidence were defined, based on Sackett et al. [16] and Ariens et al. [17] (see additional file 2, Table S2). Only significant associations were considered in this synthesis, defined by a threshold of p ≤ 0.05 or odds ratios (OR) with a confidence interval (CI) not including 1.0.

Significance of differences between groups was assessed using chi-square analysis. When not reported but sufficient data were available, the association between prognostic factors and outcome was calculated as ORs with 95% CIs.

## Results

**Figure 1:**
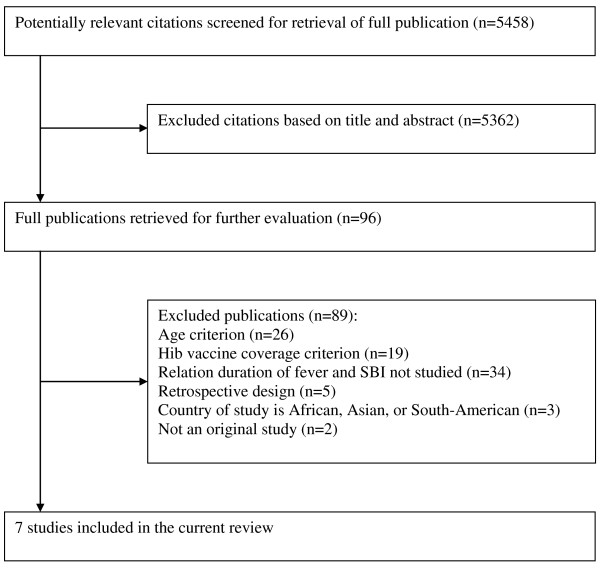
**Identification and inclusion of studies in the present review**.

The search strategy yielded 5458 citations, of which 96 could not be excluded based on title and abstract. Full-text versions of these 96 citations were retrieved. Figure 1 presents a flow chart of the process of identification and exclusion. Seven publications were included [18-24].

All seven studies were cross-sectional studies and were performed in an emergency room setting. Three studies concerned children aged 1 to 36 months [18,21,22], and two studies included children aged 3 to 36 months [20,24]. The two remaining studies concerned children aged 2 to 6 months [19], or 0 to 18 years [23]. The median or mean ages of the children in the studies were all within our two months to six years inclusion criterion.

Table 2 gives the results of the methodological quality assessment after consensus. The overall kappa before consensus was 0.73, indicating substantial agreement [15]. In all cases of initial disagreement, consensus was achieved between the two teams of reviewers. Six studies were of high quality according to our predefined criterion; the median score was 63 (range 45-74%), one study had a score of 45% [24]. The details of the included studies are given in additional file 3, Table S3. ORs were calculated using data from the studies by Pratt et al. [22].

**Table 2:** Results of quality assessment of the methodology of the included studies.

*Item (see Table 1)*	***Pulliam et al***.^***25***^	***Isaacman et al*.**^***22***^	***Fernandez Lopez et al*.**^***20***^	***Hsiao et al*.**^***21***^	***Trautner et al*.**^***27***^	***Pratt et al*.**^***26***^	***Guen et al*.**^***28***^
1	+	+	+	+	+	+	+
2	+	+	+	+	+	+	+
3	-	-	-	+	+	-	-
4	+	-	+	-	+	+	+
5	+	-	-	-	+	+	-
6	+	**+**	?	+	NA	+	?
7	NA	NA	?	+	NA	NA	NA
8	NA	NA	?	?	NA	NA	?
9	-	-	+	+	+	-	-
10	+	+	+	+	+	+	+
11	NA	NA	NA	NA	NA	NA	NA
12	-	+	+	+	+	?	+
13	+	+	+	-	+	+	-
14	+	+	+	?	-	-	?
15	+	+	?	?	+	+	+
16	-	-	-	-	-	-	?
17	+	+	+	-	-	+	+
18	+	-	-	+	-	+	-
19	-	-	-	-	-	-	-
20	+	+	+	+	+	+	+
21	+	+	+	+	+	+	+
22	+	+	-	-	-	-	-
Total score (%)	74	63	52	52	67	63	45

+; positive, -; negative, ?; unclear, NA; not applicable

### Predictive value of duration of fever for SBI

Seven studies, including a total of 1644 children, provided information on the predictive value of duration of fever at presentation for identifying SBI [18-24]. All seven studies were cross-sectional, five of which were performed in the USA [19-23], one in France [24], and one in Spain [18]. Fever was defined as a minimal temperature of 38°C [18-20] or 39°C [21,22,24]. One study investigated hyperpyrexia defined as ≥41.1°C [23]. Temperature was measured rectally [19,23], axillary [18], or at an unspecified location [20-22,24]. The definition of the outcome of SBI varied between occult bacterial infections only, and localized or invasive bacterial infections including occult bacteremia. Between and within the various studies, the diagnostic tests for SBIs (e.g. lumbar puncture) were performed in all patients or only in selected patients.

One study showed a significant univariate association of duration of fever at presentation with occult bacterial infection [19] and another study showed a significant association in a multivariate model [20]. One study of low quality provided an overall median prior duration of fever of 24 (range 0.25-192) hours versus 4.6 (± 3.13) hours in children with occult bacteremia [24]. However, no p-value or CI was provided. The remaining four studies showed no significant association, either in the univariate [18,22,23] or the multivariate analysis [21]. Therefore, according to our classification (Table 2), the level of evidence for the association between the duration of fever at presentation and a SBI is inconclusive.

## Discussion

### Summary of main findings

The predictive value of duration of fever at presentation for SBI remains contradictory and hence inconclusive.

### Strengths and limitations of this study

The number of studies in this review is relatively small, with only a few studies available for our objective. Although we initially retrieved a high number of publications using a sensitive search strategy, many studies did not fulfill our inclusion criteria. This reflects the lack of information on the duration of fever in children in the post-Hib era, making it difficult to draw firm conclusions on the duration of fever and its predictive value for SBI.

Trautner et al. showed that duration of fever is not predictive for SBI [23]. However, their study included children with hyperpyrexia only, defined as a rectal temperature of ≥41.1°C, measured at the emergency room. Thereby, their study population is not representative for patients seen in general practice. By focusing on a subgroup with hyperpyrexia, other factors may better predict SBI in this latter study population than duration of fever.

None of the studies controlled for use of antipyretics or antibiotics, which may have confounded the results of these studies.

All studies were performed in secondary and tertiary care settings. Due to selected and different study populations, the results found may not be relevant for a primary care setting. For example, in the study of Trautner et al., seven of the twenty patients with a SBI had a pre-existing condition.

### Comparison with existing literature

A recent review described the diagnostic value of clinical features to identify serious infections in children [25]; however, they included fewer and different studies addressing duration of fever or illness. We excluded four out of five studies, because they did not meet our inclusion criteria for age [26], Hib coverage [2], study design [3], and fever [27]; this makes the results of the reviews less comparable. Van den Bruel et al. [25] concluded that duration of fever or illness is not a strong predictor for serious infections, which is in line with our conclusion.

### Implications for future research or clinical practice

An explanation for the inconclusive findings for a predictive value of duration of fever might be the heterogeneity of the definition of SBI. One study reported a trend of shorter duration of fever and the possibility of bacteremia compared to the overall group [24]. Other studies, that did not meet the inclusion criterion for Hib vaccination coverage, found similar results [28,29]. It is plausible that the predictive value of the duration of fever depends on the specific SBI under study. A comparable explanation was put forward in the NICE guideline [8]. For example, bacteremia, meningitis and sepsis are SBIs that can develop relatively quickly, whereas bacterial pneumonia or urinary tract infection may develop over a longer period of time. All the other studies in our review, looking at duration of fever as predictor for SBI, included bacteremia, but they may have diluted the prognostic value of duration of fever by analyzing bacteremia combined with other SBIs. However, in general practice a broad spectrum of both slow and quick-developing SBIs will be presented. Therefore, relations other than a linear association between duration of fever and SBI may be more appropriate. Multivariate analyses considering the interaction between duration of fever and other variables (e.g. level of illness, age), and stratification for different kinds of SBIs, may yield more data about the relationship between duration of fever and risk of SBI. Observational studies are needed to test this hypothesis and thereby elucidate the duration of fever and its significance in the management of febrile children in primary care. Until then, it seems appropriate not to use duration of fever to assess the risk of SBI in febrile children in primary care.

## Conclusion

The predictive value of duration of fever at presentation for SBI remains contradictory and hence inconclusive. None of these seven studies was performed in primary care. Studies evaluating the duration of fever and its predictive value in children in primary care are required.

## List of abbreviations

SBI: serious bacterial infection; OBI: occult bacterial infection; GP: general practitioner; Hib: Haemophilus influenzae type b;

## Conflict of interest statement

The authors declare that they have no competing interests.

## Authors' contributions

GE conducted the online searches, and participated in the study selection, data extraction, quality assessment, and analyses. MM was responsible for the conduction of the online searches and study protocol. She participated in the online searches, study selection, data extraction, quality assessment, and analyses.

JCvdW supervised the design and execution of the study, and participated in the study selection, and data extraction. BWK supervised the design and execution of the study.

MYB had primary responsibility for the online searches and study protocol. She participated in the online searches, study selection, data extraction, quality assessment, and analyses. All authors contributed to the writing of the manuscript. All authors read and approved the final manuscript.

## Ethical approval

Ethical approval was not required.

## Funding body

Department of General Practice, Erasmus MC, Rotterdam.

## Pre-publication history

The pre-publication history for this paper can be accessed here:

http://www.biomedcentral.com/1471-2296/12/33/prepub

## Supplementary Material

Additional file 1 Table S1: Search strategy for Medline. Click here for file

Additional file 2 Table S2: Levels of evidence for the prognostic factors. Click here for file

Additional file 3 Table S3: Details of the included studies on the predictive value of prior duration of fever and serious bacterial infection (SBI). Click here for file

## References

[B1] Bruijnzeels MA Foets M van der Wouden JC van den Heuvel WJ Prins A Everyday symptoms in childhood: occurrence and general practitioner consultation rates Br J Gen Pract 1998 48 880 884 9604409 PMC1409911

[B2] Berger RM Berger MY van Steensel-Moll HA Dzoljic-Danilovic G Derksen-Lubsen G A predictive model to estimate the risk of serious bacterial infections in febrile infants Eur J Pediatr 1996 155 468 473 10.1007/BF01955183 8789763

[B3] Bleeker SE Derksen-Lubsen G Grobbee DE Donders AR Moons KG Moll HA Validating and updating a prediction rule for serious bacterial infection in patients with fever without source Acta Paediatr 2007 96 100 104 17187613 10.1111/j.1651-2227.2006.00033.x

[B4] Goh PL Lee SW Wong EH Predictors of serious bacterial infection in children aged 3 to 36 months with fever without source Singapore Med J 2006 47 276 280 16572237

[B5] Gorelick MH Shaw KN Clinical decision rule to identify febrile young girls at risk for urinary tract infection Arch Pediatr Adolesc Med 2000 154 386 390 10768678 10.1001/archpedi.154.4.386

[B6] Murphy CG van de Pol AC Harper MB Bachur RG Clinical predictors of occult pneumonia in the febrile child Acad Emerg Med 2007 14 243 249 10.1111/j.1553-2712.2007.tb01781.x 17242382

[B7] Berger MY Boomsma LJ Albeda FW Dijkstra RH Graafmans TA Van der Laan JR Lemmen WH Oteman N NHG-Standaard. Kinderen met koorts (Tweede herziening) Huisarts Wet 2008 51 6 287 296 in Dutch 10.1007/BF03086785

[B8] Richardson M Lakhanpaul M Guideline Development Group and the Technical Team Assessment and initial management of feverish illness in children younger than 5 years: summary of NICE guidance BMJ 2007 334 1163 1164 10.1136/bmj.39218.495255.AE 17540946 PMC1885352

[B9] World Health Organization WHO position paper on Haemophilus influenzae type b conjugate vaccines Wkly Epidemiol Rec 2006 81 445 452 (Replaces WHO position paper on Hib vaccines previously published in the Weekly Epidemiological Record) 17124755

[B10] World Health Organization WHO vaccine-preventable diseases: monitoring system 2005 global summary 2006 World Health Organization: international website http://www.who.int/vaccinesdocuments/DocsPDF05/WHO_IVB_2005.pdf

[B11] Wilczynski NL Haynes RB Developing optimal search strategies for detecting clinically sound prognostic studies in MEDLINE: an analytic survey BMC Med 2004 2 23 10.1186/1741-7015-2-23 15189561 PMC441418

[B12] Haynes RB Wilczynski NL Optimal search strategies for retrieving scientifically strong studies of diagnosis from Medline: analytical survey BMJ 2004 328 1040 10.1136/bmj.38068.557998.EE 15073027 PMC403841

[B13] Robinson KA Dickersin K Development of a highly sensitive search strategy for the retrieval of reports of controlled trials using PubMed Int J Epidemiol 2002 31 150 153 10.1093/ije/31.1.150 11914311

[B14] Hayden JA Cote P Bombardier C Evaluation of the quality of prognosis studies in systematic reviews Ann Intern Med 2006 144 427 437 16549855 10.7326/0003-4819-144-6-200603210-00010

[B15] Landis JR Koch GG The measurement of observer agreement for categorical data Biometrics 1977 33 159 174 10.2307/2529310 843571

[B16] Sackett DL Straus SE Richardson WS Rosenberg W Haynes RB Evidence-based medicine. How to practice and teach EBM 2000 Edinburgh: Churchill Livingstone

[B17] Ariens GA van Mechelen W Bongers PM Bouter LM van der Wal G Physical risk factors for neck pain Scand J Work Environ Health 2000 26 7 19 10744172 10.5271/sjweh.504

[B18] Fernandez Lopez A Luaces Cubells C Garcia Garcia JJ Fernandez Pou J Spanish Society of Pediatric Emergencies Procalcitonin in pediatric emergency departments for the early diagnosis of invasive bacterial infections in febrile infants: results of a multicenter study and utility of a rapid qualitative test for this marker Pediatr Infect Dis J 2003 22 895 903 10.1097/01.inf.0000091360.11784.21 14551491

[B19] Hsiao AL Chen L Baker MD Incidence and predictors of serious bacterial infections among 57- to 180-day-old infants Pediatrics 2006 117 1695 1701 10.1542/peds.2005-1673 16651326

[B20] Isaacman DJ Burke BL Utility of the serum C-reactive protein for detection of occult bacterial infection in children Arch Pediatr Adolesc Med 2002 156 905 909 12197798 10.1001/archpedi.156.9.905

[B21] Pulliam PN Attia MW Cronan KM C-reactive protein in febrile children 1 to 36 months of age with clinically undetectable serious bacterial infection Pediatrics 2001 108 1275 1279 10.1542/peds.108.6.1275 11731648

[B22] Pratt A Attia MW Duration of fever and markers of serious bacterial infection in young febrile children Pediatr Int 2007 49 31 35 10.1111/j.1442-200X.2007.02316.x 17250502

[B23] Trautner BW Caviness AC Gerlacher GR Demmler G Macias CG Prospective evaluation of the risk of serious bacterial infection in children who present to the emergency department with hyperpyrexia (temperature of 106 degrees F or higher) Pediatrics 2006 118 34 40 10.1542/peds.2005-2823 16818546 PMC2077849

[B24] Guen CG Delmas C Launay E Caillon J Loubersac V Picherot G Roze JC Contribution of procalcitonin to occult bacteraemia detection in children Scand J Infect Dis 2007 39 157 159 10.1080/00365540600904753 17366034

[B25] Van den Bruel A Haj-Hassan T Thompson M Buntinx F Mant D European Research Network on Recognising Serious Infection investigators Diagnostic value of clinical features at presentation to identify serious bacterial infection in children in developed countries: a systematic review Lancet 2010 375 834 845 10.1016/S0140-6736(09)62000-6 20132979

[B26] Andreola B Bressan S Callegaro S Liverani A Plebani M Da Dalt L Procalcitonin and C-reactive protein as diagnostic markers of severe bacterial infections in febrile infants and children in the emergency department Pediatr Infect Dis J 2007 26 672 677 10.1097/INF.0b013e31806215e3 17848876

[B27] Van den Bruel A Aertgeerts B Bruyninckx R Aerts M Buntinx F Signs and symptoms for diagnosis of serious infections in children: a prospective study in primary care Br J Gen Pract 2007 57 538 546 17727746 PMC2099636

[B28] Haddon RA Barnett PL Grimwood K Hogg GG Bacteraemia in febrile children presenting to a paediatric emergency department Med J Aust 1999 170 475 478 10376023 10.5694/j.1326-5377.1999.tb127847.x

[B29] Teach SJ Fleisher GR Duration of fever and its relationship to bacteremia in febrile outpatients three to 36 months old. The Occult Bacteremia Study Group Pediatr Emerg Care 1997 13 317 319 10.1097/00006565-199710000-00004 9368242

