# Supplementary material for: Duration of fever and serious bacterial infections in children: a systematic review
Source: BMC Fam Pract. 2011 May 16;12:33. doi: 10.1186/1471-2296-12-33 (PMC3111584; doi:10.1186/1471-2296-12-33)
Supplement: Additional file 1 — Table S1: Search strategy for Medline. [file 1471-2296-12-33-S1.DOC]

| 1) Prognostic studies:  fever[mesh] and (child, preschool[mesh] or infant[mesh]) and (incidence[MeSH:noexp] OR mortality[MeSH Terms] OR follow up studies[MeSH:noexp] OR prognos*[Text Word] OR predict*[Text Word] OR course*[Text Word])  2) Diagnostic studies:  fever[mesh] and (child, preschool[mesh] or infant[mesh]) and (bacterial infections[mesh] or serious bacterial infection* or infection[mesh] or hospitalization) and (sensitiv*[Title/Abstract] OR sensitivity and specificity[MeSH Terms] OR diagnos*[Title/Abstract] OR diagnosis[MeSH:noexp] OR diagnostic *[MeSH:noexp] OR diagnosis,differential[MeSH:noexp] OR diagnosis[Subheading:noexp])  3) Randomized trials:  fever[mesh] and (child, preschool[mesh] or infant[mesh]) and (randomized controlled trial [pt] OR controlled clinical trial [pt] OR randomized controlled trials [mh] OR random allocation [mh] OR double-blind method [mh] OR single-blind method [mh] OR clinical trial [pt] OR clinical trials [mh] OR "clinical trial" [tw] OR ((singl* [tw] OR doubl* [tw] OR trebl* [tw] OR tripl* [tw]) AND (mask* [tw] OR blind* [tw])) OR "latin square" [tw] OR placebos [mh] OR placebo* [tw] OR random* [tw] OR research design [mh:noexp] OR comparative study [mh] OR evaluation studies [mh] OR follow-up studies [mh] OR prospective studies [mh] OR cross-over studies [mh] OR control* [tw] OR prospectiv* [tw] OR volunteer* [tw]) NOT (animal [mh] NOT human [mh]) |
| --- |

Table S1. Search strategy for Medline
