# Supplementary material for: Duration of fever and serious bacterial infections in children: a systematic review
Source: BMC Fam Pract. 2011 May 16;12:33. doi: 10.1186/1471-2296-12-33 (PMC3111584; doi:10.1186/1471-2296-12-33)
Supplement: Additional file 2 — Table S2: Levels of evidence for the prognostic factors. [file 1471-2296-12-33-S2.DOC]

##### ***Table S2: Levels of evidence for the prognostic factors.***

| *Level of evidence* | *Definition* |
| --- | --- |
| Strong | Consistent findings (≥ 75%) in at least two high-quality studies |
| Moderate | Consistent findings (≥ 75%) in one high-quality study and at least one low-quality study |
| Limited | Findings of one high-quality study or consistent findings (≥ 75%) in at least three low-quality studies |
| Inconclusive | Inconsistent findings irrespective of study quality or less than  three low-quality studies available |
| No evidence | No data presented |
