# Supplementary material for: Duration of fever and serious bacterial infections in children: a systematic review
Source: BMC Fam Pract. 2011 May 16;12:33. doi: 10.1186/1471-2296-12-33 (PMC3111584; doi:10.1186/1471-2296-12-33)
Supplement: Additional file 3 — Table S3: Details of the included studies on the predictive value of prior duration of fever and serious bacterial infection (SBI). [file 1471-2296-12-33-S3.DOC]

Table S3: Details of the included studies on the predictive value of prior duration of fever and serious bacterial infection (SBI).

| *Author (year)* | *Design and setting* | *Inclusion criteria* | *Outcome* | *Prognostic factor* | *Results* |
| --- | --- | --- | --- | --- | --- |
| Pulliam  et al.^25^  (2001) | Cross-sectional  study  Tertiary care emergency room,  USA  n=77 | - Age 1 to 36 months  - Fever ≥ 39°C  - Clinically undetectable source of fever | Serious bacterial infection (SBI):  Occult bacteremia, urinary tract infection, pneumonia,  meningitis, septic arthritis, osteomyelitis  Diagnosis based on laboratory or radiology | Duration of fever prior to presentation  Measurement method not given, duration probably obtained by history taking | Median duration of fever in  SBI: 24 (range 3-168) h  No SBI: 24 (range 1-168) h  p=0.24  Multivariate analysis:  Duration of fever prior to presentation reported as unrelated to SBI |
| Isaacman  et al.^22^  (2002) | Cross-sectional study  Secondary care emergency room,  USA  n=256 | - Age 3 to 36 months  - Fever ≥ 39°C  - Requiring complete blood count and blood culture as part of evaluation | Occult bacterial infection (OBI):  occult pneumonia,  occult urinary tract infection,  occult bacteremia,  and no focal abnormalities on physical examination  Diagnosis based on blood or urine culture or radiology | Length of existing febrile illness in hours, not further specified, probably prior to presentation  Measurement method not given, duration recorded at emergency room, probably by history taking | Median period of febrile illness in  OBI: 24 (range 4-240) h  No OBI: 24 (range 0-288) h  Multivariate analysis:  Model 1:  Risk increase for each 1 h increase in period of febrile illness, adjusted for ANC and CRP:  OR 1.01 (95% CI 1.00-1.03, p=0.01)  Model 2:  Risk increase for each 1 h increase in period of febrile illness, adjusted for WBC and CRP:  OR 1.01 (95% CI 1.00-1.02, p=0.05) |
| Fernandez-  Lopez  et al.^20^  (2003) | Cross-sectional study  Secondary/ tertiary care emergency room,  Spain  n=445 | - Age 1 to 36 months  - Treated for fever ≥ 38°C  - Requiring blood analysis | Localized bacterial infection:  - Bacterial tonsillitis  - Peritonsillar abscess  - Acute otitis media  - Mastoiditis  - Gastroenteritis in children aged > 3 months  - Lower urinary tract infection  Invasive bacterial infection:  - Meningitis  - Sepsis  - Bone/joint infection  - Acute pyelonephritis  - Lobar pneumonia  - Bacterial enteritis, age < 3 months  - Occult bacteremia  Diagnosis based on culture or rapid test or radiology or otorhinolaryngologist | Evolution of fever time, in hours, not further specified, probably prior to presentation  Measurement method not given, duration probably obtained by history taking | Evolution of fever time in  Viral infection:  36.2 ± 42.5 h  Bacterial infection:  37.1 ± 43.7 h  Invasive bacterial infection:  41.2 ± 47.2 h  Noninvasive infection:  33.3 ± 39.6 h  Univariate analysis:  Mean evolution of fever time compared between the groups was not significantly different |
| Hsiao  et al.^21^  (2006) | Cross-sectional study  Tertiary care emergency room,  USA  n=429 | - Age 57 to 180 days  - Rectal temperature > 37.9°C | SBI:  Bacteruria  Bacteremia  Diagnosis based on urine or blood culture;  Final diagnosis from computerized hospital records | Duration of fever before evaluation  Measurement method not given, duration probably obtained by history taking | Duration of fever in  SBI: 26.5 ± 41.5 h  No SBI: 18.6 ± 21.7 h  Univariate analysis:  Duration of fever before evaluation was significantly longer in infants with SBI compared with those without (p<0.001) |
| Trautner et al.^27^  (2006) | Cross-sectional study  Tertiary care emergency room, USA  n=103 | - Age <18 years  - Oral, axillary, or ear temperature >40°C, and a rectal temperature > 41.1°C | SBI: growth of a clinical significant bacterial pathogen from blood, urine, stool, cerebrospinal fluid, or any normally sterile body site | Duration of fever before presentation categorized in <24 h, 24-48 h, and >48 h  Measurement method not given, duration probably obtained by history taking | Risk of SBI predicted by fever:  - <24 h: 40% OR: reference group  - 24-48 h: 15% OR 0.30 (95% CI 0.07-1.26)  - >48 h: 45% OR 1.04 (95% CI 0.35-3.12)  Univariate analysis: duration of fever is not predictive of SBI rather than viral infection. |
| Pratt et al.^26^ (2007) | Cross-sectional study  Tertiary care emergency room, USA  n=119 | - Age 1-36 months  - Fever documented or reported > 39°C | SBI: bacteremia, meningitis, urinary tract infection, pneumonia, septic arthritis, and osteomyelitis  Diagnosis based on:  - Bacteremia: recovery of a single bacterial pathogen using standard culture techniques  - Urinary tract infection: growth of a single urinary tract pathogen at >10^4^ c.f.u./mL on a catheterized specimen  - Pneumonia: presence of a local infiltrate on chest X-ray as interpreted by the pediatric radiologist | Duration of fever at presentation <12 h or >12 h  Measurement method not given, duration probably obtained by history taking | < 12 h fever and SBI: 13%  >12 h fever and SBI: 15%  OR 0.881 (95% CI: 0.302-2.574)  Univariate analysis:  No significant difference between the SBI positive and negative group within the <12 h and >12 h groups when compared by duration of fever |
| Guen et al.^28^ (2007) | Cross-sectional study  Tertiary care emergency room, France  n=215 | - Age 3-36 months  - Unexplained fever of >39°C documented in the emergency department or at home. | SBI: bacteremia  Diagnosis based on:  Positive blood culture.  (the following cultures were considered as contaminants: Corynebacteria, Staphylococcus epidermidis, Staphylococcus hominis, Staphylococcus capitis, Bacillus sp., Streptococcus mitis) | Duration of fever at presentation in hours  Measurement method not given, duration probably obtained by history taking | Duration of fever:  Overall median: 24 (range 0.25-192) h  SBI: 4.6 (+ 3.13) h |
